# Computational Design and Evaluation of Peptides to Target SARS-CoV-2 Spike–ACE2 Interaction

**DOI:** 10.3390/molecules30081750

**Published:** 2025-04-14

**Authors:** Saja Almabhouh, Erika Cecon, Florence Basubas, Ruben Molina-Fernandez, Tomasz Maciej Stepniewski, Jana Selent, Ralf Jockers, Amal Rahmeh, Baldo Oliva, Narcis Fernandez-Fuentes

**Affiliations:** 1 Structural Bioinformatics Laboratory (GRIB-IMIM), Department of Medicine and Life Sciences, Universitat Pompeu Fabra, 08003 Barcelona, Spain; sajash.almabhouh@upf.edu (S.A.); ruben.molina-fernandez@upf.edu (R.M.-F.); baldo.oliva@upf.edu (B.O.); 2 Institute Cochin, INSERM, CNRS, Université Paris Cité, F-75014 Paris, France; erika.cecon@inserm.fr (E.C.); ralf.jockers@inserm.fr (R.J.); 3 Synthetic Biology, Department of Medicine and Life Sciences, Universitat Pompeu Fabra, 08003 Barcelona, Spain; fbasubas@bistgraduatecentre.com (F.B.); amal.rahmeh@upf.edu (A.R.); 4 GPCR Drug Discovery, Hospital del Mar Research Institute, 08003 Barcelona, Spain; tm.stepniewski@gmail.com (T.M.S.); jana.selent@upf.edu (J.S.); 5 Institute of Biological, Environmental and Rural Sciences (IBERS), Aberystwyth University, Aberystwyth SY23 3EE, UK

**Keywords:** peptide design, SARS-CoV-2, spike protein, ACE2 receptor, molecular dynamics, time-resolved FRET assay, pseudotyped viral particles

## Abstract

The receptor-binding domain (RBD) of SARS-CoV-2 spike protein is responsible for the recognition of the Angiotensin-Converting Enzyme 2 (ACE2) receptor in human cells and, thus, plays a critical role in viral infection. The therapeutic value of targeting this interaction has been proven by a sizable body of research investigating antibodies, small proteins, aptamers, and peptides. This study presents a novel peptide that impinges the interaction between RBD and ACE2. Starting from a very large pool of structurally designed peptides extracted from our database, PepI-Covid19, a diverse set of peptides were studied using molecular dynamics simulations. Ten of the most promising were chemically synthesized and validated both in vitro and in a cell-based assay. Our results indicate that one of the peptides (PEP10) exhibited the highest disruption of the RBD/ACE2 complex, effectively blocking the binding of two molecules and consequently inhibiting the SARS-CoV-2 spike-mediated cell entry of viruses pseudotyped with the spike of the D614G, Delta, and Omicron variants. PEP10 can potentially serve as a scaffold that can be further optimized for improved affinity and efficacy.

## 1. Introduction

The COVID-19 pandemic has profoundly impacted global health, highlighting the urgent need for effective therapeutics against SARS-CoV-2, the virus responsible for the disease. The spike protein of SARS-CoV-2, specifically its Receptor-Binding Domain (RBD), plays a crucial role in mediating viral entry into host cells by interacting with the Angiotensin-Converting Enzyme 2 (ACE2) receptor [1]. Given the critical nature of this interaction, targeting the RBD-ACE2 interface represents a promising strategy for inhibiting viral infection and mitigating the spread of the virus.

Much research has been devoted to targeting the interaction of RDB-ACE2 for therapeutic interventions that can effectively disrupt this interface. As shown in recent extensive reviews, this includes the development of antibodies [2], nanobodies [3], and mini-proteins and peptides [4]. Significant progress has been made in the development of novel potential therapies; however, challenges persist given the rapid evolution of SARS-CoV-2 [5,6]. In particular, mutations in the RBD, which have been widely documented [7,8,9], can compromise the effectiveness of existing therapeutics, demonstrating the need for novel agents.

Structure-based peptide design has emerged as a potent approach for developing inhibitors targeting protein–protein interactions [10]. The accumulation of structural data on SARS-CoV-2, with over 4500 entries in the Protein Data Bank (PDB) [11], helps in identifying potential peptide candidates. This wealth of information led to the creation of our PepI-Covid19 database [12], which catalogues designed peptides based on their predicted binding affinities and structural characteristics.

In this study, we explore the use and evaluation of peptides selected from the PepI-Covid19 database to target the RBD of SARS-CoV-2, assessing their binding efficiency through molecular dynamics simulations and determining their inhibitory effects in vitro and using a cell-based assay. As mentioned earlier, the use of peptides is a valid approach to complement existing therapeutic avenues, and this work seeks to contribute valuable insights to the development of therapeutic strategies against SARS-CoV-2. We leverage targeted peptide design to exploit the large repertoire of structural information available as a complementary avenue to develop novel agents against COVID-19.

Currently, the PepI-Covid19 database contains over one million designed peptides derived from six different RBD protein complexes. Through a set of stringent filters, highly structurally diverse designed peptides were selected. We conducted molecular dynamics simulations to assess the binding kinetics of peptides and to select the ten most promising candidates. These candidates were synthesized and assessed as inhibitors of the RBD–ACE2 interaction both in vitro and in a cell-based assay. Our results show that one of the candidates, PEP10, interfered with the interaction between both receptors. Most importantly, PEP10 inhibited the cell entry of viruses pseudotyped with the spike receptor of the D614, Delta, and Omicron variants.

## 2. Results and Discussion

### 2.1. Selection of Peptides from PepI-Covid19 Database

The peptides used in this study were selected from the PepI-Covid19 database. The PepI-Covid19 database currently contains many designed peptides derived from the structure of RBD bound to the native receptor, i.e., ACE2, and also bound to nanobody and monoclonal antibodies. Each peptide classified in the PepI-Covid19 database is characterized by a predicted binding energy as per the Rosetta energy score [13], including a reweighted score specific for protein–peptide interactions, as described in [14]. Moreover, it includes several features describing the interface, including (i) the surface area of the protein–peptide interface (Ang^2^) and (ii) the number of hydrogen bonds, including unsatisfied bonds at the interfaces (e.g., donor/acceptor groups not forming hydrogen bonds) and packing and peptide scores, as described in [15]. These features were used to make an initial selection of 20 peptides (Table 1).

**Figure 1:**
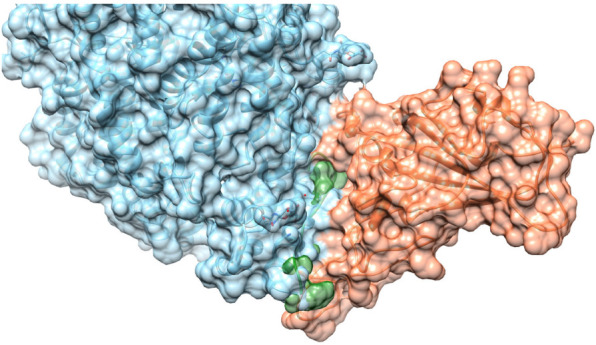
The interface between ACE2 (blue) and the RBD (orange) with PEP10 (green) docked in a pose that would interfere with their interaction. Each molecule is represented with a solvent-accessible surface, highlighting the spatial arrangement and potential interaction regions.

The main driver of this selection was the maximization of the conformational diversity of the peptides and the coverage of the native interface between RBD and ACE2, i.e., the peptides were targeting different regions of the interface. As shown in Table 1, the secondary structure of the designed peptides was also quite diverse, with half of them showing a mainly extended conformation (PEP1 to PEP10), with the rest predominantly having a high content of helical conformation (PEP11 to PEP20). As previously reported, the dominating element in the interaction between RBD and ACE2 is an alpha helix called the H1 helix [16,17]. Thus, it was very important to include peptides displaying this conformation. The coordinates of the models are shown in the Supplementary Material. Figure 1 illustrates the interface between ACE2 (blue) and the RBD (orange) with PEP10 (green) modeled to interfere with their interaction.

### 2.2. Assessment of Peptide/RBD Interactions Using MD

Molecular Dynamics (MD) simulations were performed to evaluate the binding stability and conformational dynamics of the selected peptides when interacting with the SARS-CoV-2 RBD. This approach is commonly used to predict the dynamic behavior of peptide inhibitors, with previous studies demonstrating that stable interactions observed in MD simulations often correlate with experimental efficacy [18,19,20]. Each peptide was simulated in triplicate, and the binding interactions were assessed across simulation frames.

**Figure 2:**
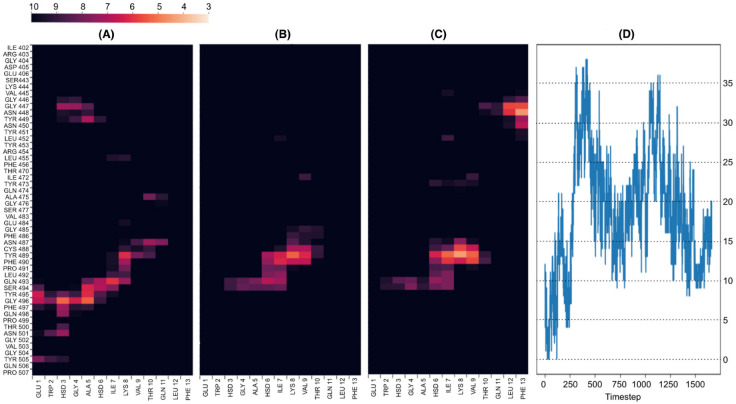

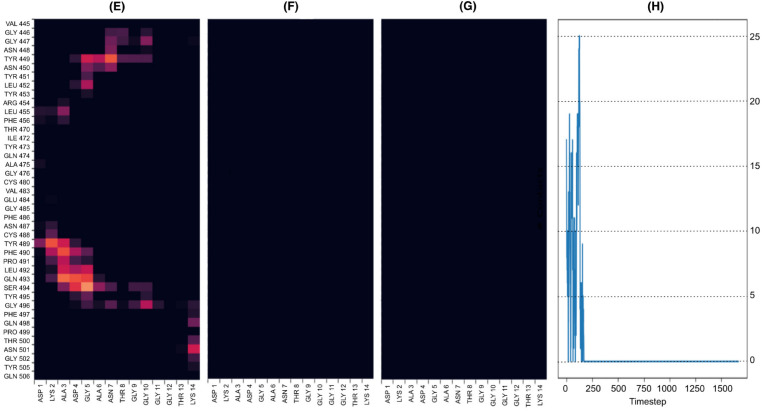
Interactions of PEP10 and PEP6 with the RBD region observed in (MD) simulations. The figure summarizes the interaction dynamics between each peptide and the RBD of SARS-CoV-2 based on MD simulation data. The top row corresponds to the persistent replicates of PEP10, while the bottom row represents one of the three non-persistent replicates of PEP6. (**A**–**C**,**E**–**G**) Distances (Å) between the α carbons of the peptide and the α carbon of the RBD, focusing only on residues with distances less than 10 Å. (**A**,**E**) Initial distances (Å) between the α carbons of the peptide and the RBD residues at the start of the simulation. (**B**,**F**) Average distances (Å) over the simulation trajectory. (**C**,**G**) Final distances (Å) at the end of the simulation. The color gradient represents the distance between α carbons, with warmer colors (red to yellow) representing closer interactions (distances < 10 Å). (**D**,**H**) The time evolution of the number of contacts between the Cα of the peptide and the RBD along the simulation timeline, with a threshold distance of 7 Å used to define a contact.

The simulations showed that peptides PEP16 and PEP17 remained attached to the interface of the RBD, forming stable interactions with the RBD in all simulation replicas. PEP10 (Figure 2A–D), PEP11, PEP12, PEP13, and PEP19 also consistently bound to the interface, but not in all replicas. Indeed, the simulations of the peptides indicated a variable binding behavior, resulting in either the dissociation of the peptides or a transition to alternative sites along the simulation. In contrast, the simulation of PEP6 (Figure 2E–H), PEP3, and PEP8 showed a detachment of the peptides earlier in the simulation and in all replicas. Based on the MD analyses, nine peptides that demonstrated binding in at least one replicate were chosen for subsequent experimental validation. These were PEP 2, 5, 10, 11, 12, 13, 16, 17, and 19 (Table 1). Additionally, PEP 1 was included as a negative control, as it did not exhibit binding. Figures depicting different replicates for all peptides are provided in the Supplementary Material (Figure S1).

### 2.3. Experimental Validation Using TR-FRET

To experimentally validate the binding efficiency of the selected peptides, we employed the Time-Resolved Förster Resonance Energy Transfer (TR-FRET) assay. This assay relies on energy transfer between the fluorescent-labeled ACE2 and d2-labeled RBD tracer, which only occurs if the two proteins are in close proximity (10 nm). This assay enabled us to quantitatively assess the inhibitory effects of various peptides on the interaction between the RBD and ACE2 [21]. This method was demonstrated to effectively monitor SARS-CoV-2 spike–ACE2 interactions in living cells and to characterize compounds impacting this interaction [22,23,24]. Among the peptides tested, PEP10 exhibited a significant binding affinity to the pike RBD, confirming its potential as an effective inhibitor of the RBD–ACE2 interaction.

**Figure 3:**
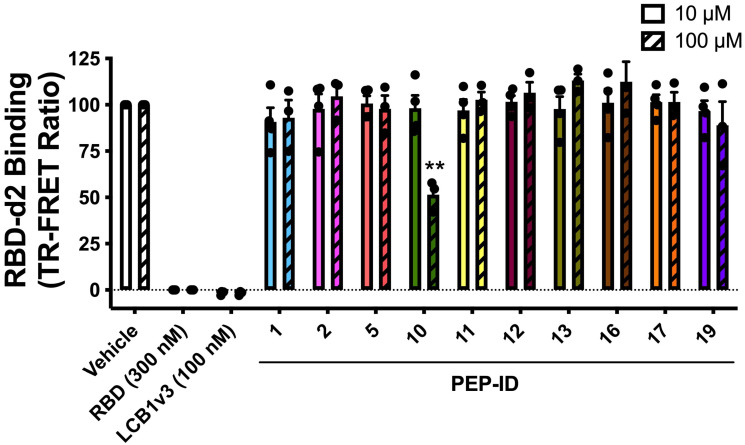
The inhibitory effects of peptides at different concentrations on spike (RBD) binding to ACE2, evaluated using TR-FRET. This figure illustrates the inhibitory effects of different peptides at two concentrations in blocking the RBD–ACE2 interaction. PEP10 at 100 µM stands out as a strong inhibitor, significantly reducing binding, making it a promising candidate for further studies. Each tested peptide is shown in a different color, with error bars representing mean and standard deviation values and dots representing the values of each individual measurement. (**) indicates a *p*-value < 0.001.

HEK293 cells expressing the SNAP-ACE2 receptor were treated with a series of peptides at two concentrations (10 µM and 100 µM), and the binding of RBD-d2 to ACE2 was measured using TR-FRET (Figure 3). Each experimental condition was performed in triplicate, with consistent results across all replicates, confirming the reproducibility of the selected peptides’ inhibitory effects.

As shown in Figure 3, most peptides displayed minimal to no inhibitory effect, with the TR-FRET ratios being comparable to that of the vehicle control. However, PEP10 demonstrated significant disruption of the RBD–ACE2 interaction at both concentrations, with a marked reduction in the TR-FRET signal, particularly at 100 µM, where inhibition was significant at *p* < 0.001 compared to the control group. While PEP10 significantly reduced the TR-FRET signal, its inhibitory effect was observed alongside the positive control LCB1v3 [25], a mini-protein known for its potent ability to block the RBD–ACE2 interaction and included as a reference.

The results reinforce the findings from the peptide selection process, demonstrating the effectiveness of PEP10 and suggesting further exploration regarding its potential role in disrupting the SARS-CoV-2 infection pathway.

### 2.4. Effect of PEP10 on SARS-CoV-2 Spike-Mediated Viral Infection

**Figure 4:**
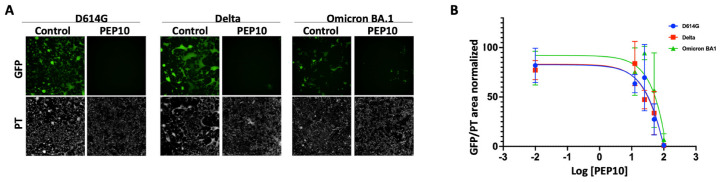
Effects of PEP10 on infectivity of SARS-CoV-2 spike pseudotyped viruses. (**A**) Representative images of the infection of Vero-TMPRSS2 cells with rVSV-GFP-∆G-Spike variants (D614G, Delta, and Omicron BA.1) with and without adding 100 µM PEP10. (**B**) Dose–response curve of PEP10 on infection on different spike variants. Viral infection was expressed as ratio of sum of intensity of GFP/area of phase transition. Data were normalized to no peptide control and expressed as mean ± S.E.M. of 3 independent experiments. IC50 was estimated using non-linear fit of log inhibitor vs. normalized response (three parameters).

To examine the effect of PEP10 on spike function in virus entry into the cell and virus spread, we used replicative recombinant vesicular stomatitis virus pseudotypes where the VSV glycoprotein G was deleted and replaced by the SARS-CoV-2 spike in a VSV genome expressing GFP (rVSV-GFP-∆G-Spike). The pseudotypes included the spike variants from SARS-CoV-2 D614G, Delta, and Omicron B.A1. Vero cells naturally expressing ACE-2 and stably overexpressing TMPRSS2 (Vero-TMPRSS2) were infected with rVSV-GFP-∆G-Spike in the vehicle control or in the presence of increasing concentrations of PEP10 (0–100 µM), and viral infection was monitored and quantified for GFP expression using high content imaging (Figure 4). PEP10 inhibited infection by viruses pseudotyped with D614G, Delta, and Omicron B.A1 spike with IC50 values of 46, 47, and 140 µM, respectively.

Our peptide, PEP10, exhibited IC_50_ values ranging from 46 to 140 μM against SARS-CoV-2 variants. While certain peptide inhibitors, such as the EK1-C16 lipopeptide (IC_50_ = 0.11–0.43 μM) [26] and D-peptides (~5–6 μM) [27], show greater potency, these inhibitors often require extensive modifications, such as lipid conjugation or unnatural peptide backbones, to enhance their efficacy. In contrast, PEP10 was designed using a natural peptide scaffold, making it a promising candidate for further optimization. Future modifications, such as residue substitutions or modifications, could improve its affinity while maintaining favorable stability and specificity.

### 2.5. Structural Analyses of PEP10

**Figure 5:**
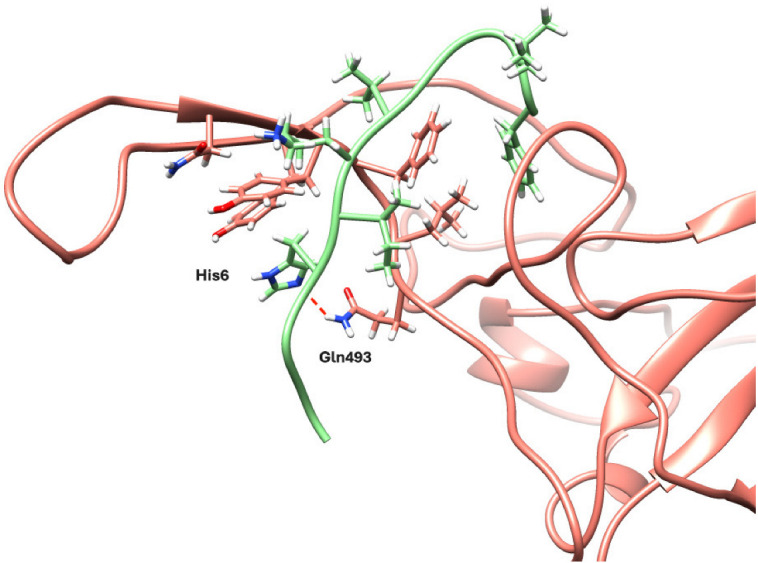
The final frame of the MD simulation showing the interaction between peptide 10 (in green) and the receptor-binding domain (RBD) (in salmon) in a cartoon representation. A preserved hydrogen bond (red dashed line) between the Gln493 residue on the RBD and the His 6 on PEP10 is highlighted with a red dashed line. Hydrophobic/aromatic interactions between nonpolar side chains of the RBD (e.g., Tyr489, Phe490, and Leu492) and PEP10 (e.g., Ala5, and Ile7) are also illustrated, suggesting stabilizing forces in the peptide–receptor complex. Selected residues are shown in stick representation where oxygen, nitrogen, and hydrogen atoms are shown in red, blue, and white, respectively; carbon atoms are shown in the same color as the PEP10 or RBD, respectively.

As shown in the in vitro validation and cell-based assay, PEP10 targeted the interaction between the RBD of the spike protein for SARS-CoV-2 and ACE2. The structural model shows that the peptide binds across the interaction interface (Figure 5), suggesting that the binding of the peptide will cause steric hindrance, preventing the recognition of ACE2. To further investigate the binding stability and identify key interacting residues, snapshots from the final frame of the MD simulation were analyzed.

Figure 5 illustrates these structural interactions, highlighting the peptide’s role in disrupting RBD-ACE2 binding. We observed some residues in the RBD with a significant interaction, including Tyr489, Phe490, Leu492, Gln493, and Glu484, which consistently interacted with residues on PEP10 (His6, Gly4, Ala5, and Ile7). These interactions suggest a stable binding interface, particularly involving some hydrogen bonding (between His6 and Gln493) and hydrophobic/aromatic contacts (between Ile7, Leu12, Phe13, and Phe490). Additional interactions were observed with RBD residues Asn487, Tyr473, and Gly447, highlighting slight shifts in binding orientation during the simulation. Notably, residues Leu12 and Phe13 on PEP10 also engaged in hydrophobic/aromatic interactions in the final frame, suggesting additional stabilizing contacts at later stages of binding.

## 3. Materials and Methods

### 3.1. Selection of Peptides from PepI-Covid19 Database

The selection of peptides followed a multistep approach, as previously described [28], with the exception of filtering out peptides with helical conformation. First, peptides were ranked based on their Rosetta energy scores, which estimate stability and binding potential. Following this, peptides were prioritized for their ability to mimic critical residues at the RBD–ACE2 interface, specifically targeting hot-spot residues identified by PCRPi [29] and interface packing efficiency. Additional factors such as hydrogen bonding capacity and sequence diversity were also considered, culminating in a final selection of 20 peptides, as shown in Table 1.

### 3.2. Molecular Dynamics Simulations

#### 3.2.1. System Preparation and Equilibration

MD simulations were employed to analyze the structural stability and interaction dynamics between the selected peptides and the RBD. The system was prepared using a Charmm-GUI input generator [30,31,32] with default parameters, which generated the necessary input files for equilibration in NAMD2 v2.14 [33].

For system preparation, the structural models of peptides bound to RBD were used as the starting model. The protein–peptide complex was then solvated in a 10 nm cubic box filled with TIP3P water molecules, and the system was neutralized by adding 0.15 M of KCl ions. The parameters for the system were derived from the Charmm36M forcefield [34].

During the equilibration step, the system temperature and pressure were maintained at 300 K and 1 atm, respectively. The temperature was coupled using the algorithm of the Nose–Hoover thermostat with a temperature coupling value of 20.05. Pressure was coupled with the Parrinello–Rahman barostat algorithm using τp = 5 and isotropic conditions. For electrostatic interactions, the Particle Mesh Ewald (PME) method [35] was used to evaluate long-range electrostatic interactions, and LINCS constraints [36] were applied to all bonds.

#### 3.2.2. Production Runs

Production runs were performed using the AceMD engine v.3.2.3 [37], with each system simulated for 1 µs in triplicate under an NVT ensemble. The temperature was maintained at 310 K using the Langevin thermostat, while hydrogen bonds were constrained via the RATTLE algorithm.

Non-bonded interactions were truncated at 9 Å with a smooth switching function applied at 7.5 Å. A timestep of 4 fs was used, enabled by a hydrogen mass repartitioning scheme implemented within AceMD.

### 3.3. Time-Resolved Fluorescence Energy Transfer Analyses

#### 3.3.1. Peptides

Peptides were purchased from the Proteomics and Protein chemistry unit at the University Pompeu Fabra. Synthesis was performed in C-terminal carboxamide form using Fmoc solid-phase peptide synthesis on an H-Rink Amide-ChemMatrix resin of a 0.50 mmol/g substitution (PCAS BioMatrix, Quebec, QC, Canada) at a 0.05 mmol scale in a Prelude automated synthesizer (Protein Technologies, Tucson, AZ, USA). Side chains of trifunctional residues were protected with TFA-labile t-butyl (Asp, Glu, Ser, Thr), trityl (Asn, Gln, His), Boc (Lys), and 2,2,4,6,7-pentamethyldihydrobenzofuran-5-sulfonyl (Arg) groups. Couplings were systematically performed with a 5-fold excess of Fmoc amino acid in the presence of *N*,*N*,*N*′,*N*′-tetramethyluronium hexafluorophosphate (5 eq) and *N*,*N*-diisopropylethylamine (10 eq), with DMF used as the solvent. Fmoc removal was performed with piperidine/DMF (20:80 *v*/*v*), followed by DMF washes. Cleavage and deprotection of the peptide resins were performed with TFA/water/triisopropylsilane (95:2.5:2.5, *v*/*v*/*v*, 90 min, r.t.). Peptides were isolated via precipitation with cold diethyl ether and centrifugation and then solubilized in water and lyophilized.

The synthetic crude products were analyzed using RP-HPLC and LC-MS and purified using preparative RP-HPLC. Analytical RP-HPLC was performed using an LC-20AD instrument (Shimadzu, Kyoto, Japan) fitted with a Luna C18 column (4.6 mm × 50 mm, 3 μm; Phenomenex, Torrance, CA, USA), using linear gradients of solvent B (0.036% TFA in ACN) into A (0.045% TFA in H_2_O) over 15 min at a 1 mL/min flow rate with UV detection at 220 nm. Preparative RP-HPLC was performed using an LC-8 instrument (Shimadzu) fitted with a Luna C18 column (21.2 mm × 250 mm, 10 μm; Phenomenex), using linear gradients of solvent D (0.1% TFA in ACN) into C (0.1% TFA in H_2_O) over 30 min with a flow rate of 25 mL/min. MS analysis was performed using an LC-MS 2010EV instrument (Shimadzu) fitted with an XBridge C18 column (4.6 mm × 150 mm, 3.5 μm, Waters, Cerdanyola del Vallès, Spain), eluting with linear gradients of F (0.08% formic acid (FA) in ACN) into E (0.1% FA in H_2_O) over 15 min at a 1 mL/min flow rate. Fractions of >95% HPLC purity and with the expected mass according to LC-MS were pooled and lyophilized.

#### 3.3.2. TR-FRET

In this TR-FRET binding assay, HEK293 cells were first transfected to express the SNAP-tagged ACE2 receptor. After 48 h, the cells were labeled with the SNAP Lumi4-Tb fluorophore substrate for one hour at 4 °C. Once labeled, the cells were washed, collected, and distributed into a 384-well plate at a density of 10 µL per well. Competitors, including peptides, LCB1v3 (used as a positive control and a benchmark for inhibition), and controls, were then added to each well in 2 µL volumes at the specified final concentrations. Following this, 2 µL of the RBD-d2-labeled spike protein was added to each well, with a final concentration of 5 nM. After a 2 h incubation period at room temperature, the TR-FRET signal was read using an Envision plate reader (PerkinElmer, Waltham, MA, USA). The signal was read again after 4 h of incubation. All treatments were performed in triplicate, and the data were expressed as the TR-FRET ratio of donor-to-acceptor fluorescence. To pool the results from three independent experiments (100 µM) or four independent experiments (10 µM), the data from each experiment were normalized, with the control group (vehicle-treated) representing 100%. Statistical analysis was performed using a one-way ANOVA, with ** *p* < 0.001 indicating significant inhibition of binding by the tested peptides. PEP10 demonstrated significant inhibition.

### 3.4. Infection by SARS-CoV-2 Spike Pseudotyped Viruses

Replicative recombinant vesicular stomatitis virus pseudotypes (rVSV-GFP-∆G-Spike) for spike variants from SARS-CoV-2 D614G, Delta, and Omicron B.A1 were gifted by Dr. Sean Whelan (University of St. Louis, MO, USA). rVSV-GFP-∆G-Spike viruses were preincubated with various concentrations of PEP10 (0–100 µM) at 37 °C for 1 h. The virus/peptide mixture was added to Vero-TMPRSS2 cells growing in 96-well plates at an MOI value of 0.01. After 1 h of incubation, the mixture was removed and replaced by media containing the corresponding concentration of peptides. GFP expression in cells was measured at 24 h using high content imaging (Operetta, PerkinElmer, Waltham, MA, USA), and the images were analyzed using the Harmony software v4.9 (PerkinElmer). Viral infection was expressed as the ratio of the sum of intensity of GFP/area of phase transition.

## 4. Conclusions

In this study, we investigated the ability of computationally designed and selected peptides to inhibit the binding interaction between the SARS-CoV-2 spike protein’s receptor-binding domain (RBD) and the ACE2 receptor—a key initial step in the viral entry process. By utilizing molecular dynamics (MD) simulations alongside TR-FRET binding and cell-based (Vero-TMPRSS2/pseudotyped viruses) assays, we identified PEP10 as a potent candidate. PEP10 demonstrated stable binding interactions with critical residues on the RBD, highlighting its significant potential to disrupt the RBD–ACE2 interaction.

The MD simulations provided detailed insights into the conformational stability and binding dynamics of PEP10, underscoring consistent interactions with key residues on the RBD over time. These computational findings were further supported by experimental validation, which revealed that PEP10 exhibits substantial inhibitory activity at micromolar concentrations, demonstrating that PEP10 exhibits substantial inhibitory activity. This efficacy at low concentrations strengthens PEP10’s potential as a lead candidate for antiviral development, with possible applications that are both potent and resource-efficient.

Notably, our study employed a SARS-CoV-2 spike pseudotyped viral infection assay, using rVSV-GFP-∆G-Spike pseudoviruses for D614G, Delta, and Omicron BA.1 variants. This setup allowed us to evaluate PEP10’s efficacy in blocking SARS-CoV-2 spike-mediated cell entry and spread. PEP10 demonstrated robust efficacy in reducing viral entry, highlighting its therapeutic potential. This promising in vitro performance suggests strong potential for PEP10 in future in vivo applications, reinforcing its viability as a candidate for antiviral development.

Moving forward, further studies should focus on enhancing PEP10’s stability, exploring structural modifications to improve its inhibitory potency, and assessing its efficacy in in vivo models. These efforts could pave the way for PEP10 to serve as a foundation for future antiviral therapeutics targeting COVID-19. Our findings demonstrate the significant potential of computationally designed peptides as molecular tools for generating anti-viral therapeutics blocking SARS-CoV-2 entry pathways.

## Figures and Tables

**Table 1:** List of peptides used in this study.

ID	Sequence	Secondary Structure ^1^	Size ^2^
PEP1 *	QDGRDDETKHED	CCHHHHHHTTCC	12
PEP2 *	QASSLDSAHWRDLYGEYY	CCGGGGGSCHHHHSCSCC	18
PEP3	TLNRGLDESSREHHRE	CCCSSCCHHHHHHHHC	16
PEP4	DEDKERHEKEDYDNQK	CHHHHHHHHTTSTTTC	16
PEP5 *	TRDKYRFGESEYED	CTTTTCTTSHHHHC	14
PEP6	DKADGANTGGGGTK	CCCCCCSSCCCCCC	14
PEP7	DKYWHQWEDERHSGGQ	CHHHHHHHHTTSSTTC	16
PEP8	GKGHTSTGTTQ	CCSCCCCCCCC	11
PEP9	GGGQSSTGRGKD	CCCCCEECTTCC	12
PEP10 *	EWHGAHIKVTQLF	CCSSCCCCCCCCC	13
PEP11 *	QDDTQEDKDRHLKDEIYK	CHHHHHHHHHHHHHHHHC	18
PEP12 *	QRFSEERYRAWVSHEND	CCCCHHHHHHHHHHHTC	17
PEP13 *	QLGDLHRDRKGEENNRQ	CCCCCCHHHHHHHHHHC	17
PEP14	QEQTERDKRQHEKDSDWYQ	CHHHHHHHHHHHHHHHSCC	19
PEP15	TDEDKKYH	CHHHHHHC	8
PEP16 *	TDAGKDGWADHIYHRQY	CGGGGGGHHHHHHHHHC	17
PEP17 *	TSDDFAEEHWKAHAGAYKL	CHHHHHHHHHHHTHHHHHC	19
PEP18	NEDKNRHGEASYGNQYG	CHHHHHHHHHTTCCCCC	17
PEP19 *	DAERRREEEKGRDQ	CHHHHHHHHHTTCC	14
PEP20	AAEEQRKRDEWWRKGTS	CHHHHHHHHHHHHTTCC	17

^1^ C: Coil; H: Helical; E: Extended Beta; G: 3^10^ Helix; T: Turn; S: Bend. ^2^ Number of residues. * Peptides selected for TR-FRET assay.

## Data Availability

The original contributions presented in this study are included in the article and Supplementary Materials. Further inquiries can be directed to the corresponding author.

## Supplementary Materials

The following supporting information can be downloaded at https://www.mdpi.com/article/10.3390/molecules30081750/s1, File: models_coordinates.tar. A tar-compressed file containing the atomic coordinates of the PEP1 to PEP20 RBD–peptide complexes. File: Supplementary_FigS1.pdf. A PDF file containing Supplementary Figure S1.
