# Supplementary material for: Computational Design and Evaluation of Peptides to Target SARS-CoV-2 Spike–ACE2 Interaction
Source: Molecules. 2025 Apr 14;30(8):1750. doi: 10.3390/molecules30081750 (PMC12029774; doi:10.3390/molecules30081750)
Supplement: Supplementary file 1 [file molecules-30-01750-s001.zip › Supplementary_FigS1.pdf]

A

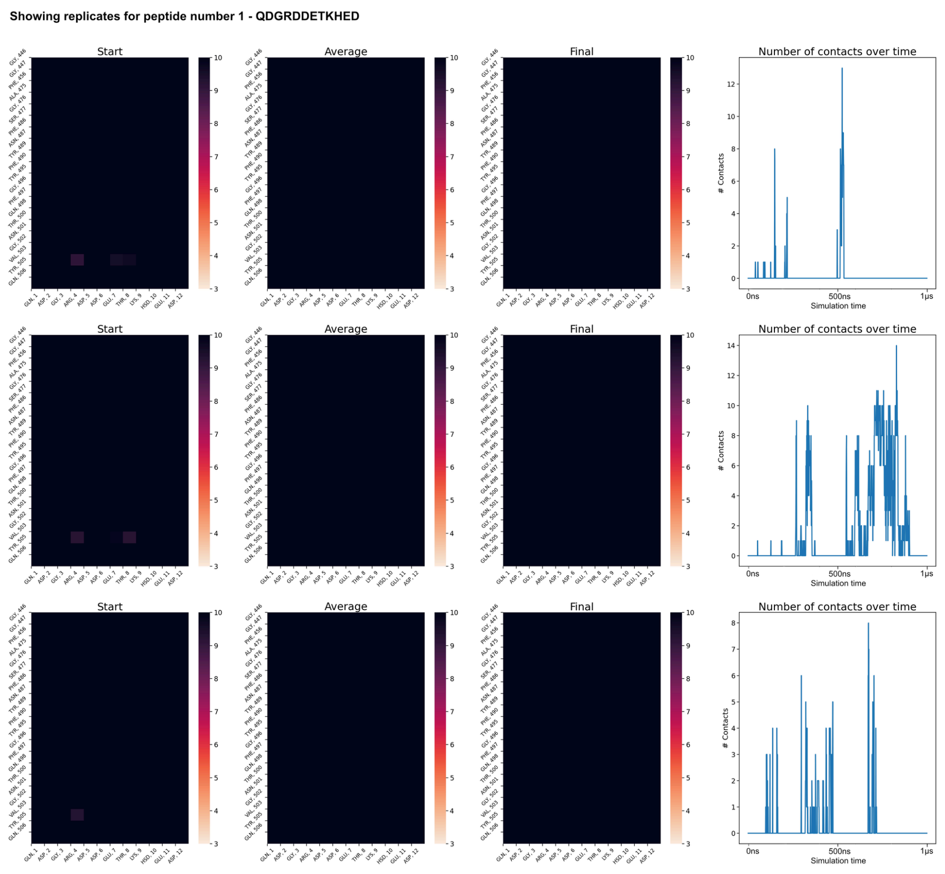
Showing replicates for peptide number 1 - QDGRDDETKHED
Start
Average
Final
Number of contacts over time
Simulation time

B

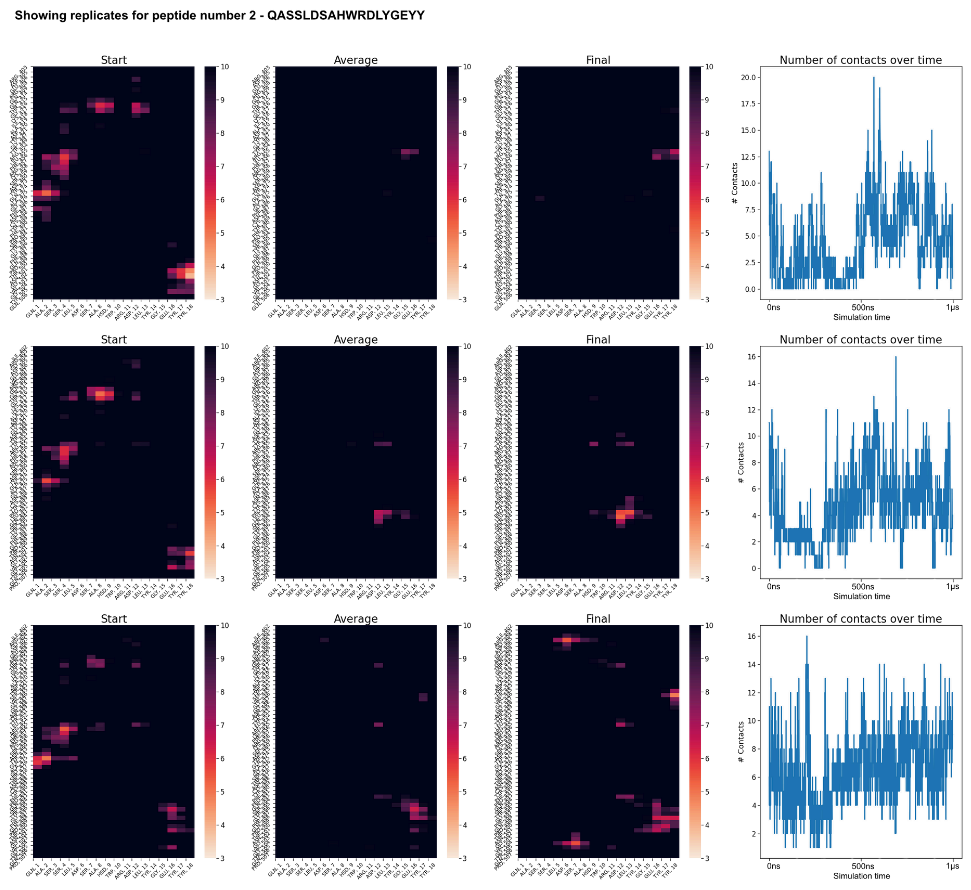
Showing replicates for peptide number 2 - QASSLDSAHWRDLYGEYY
Start
Average
Final
Number of contacts over time
Simulation time

C

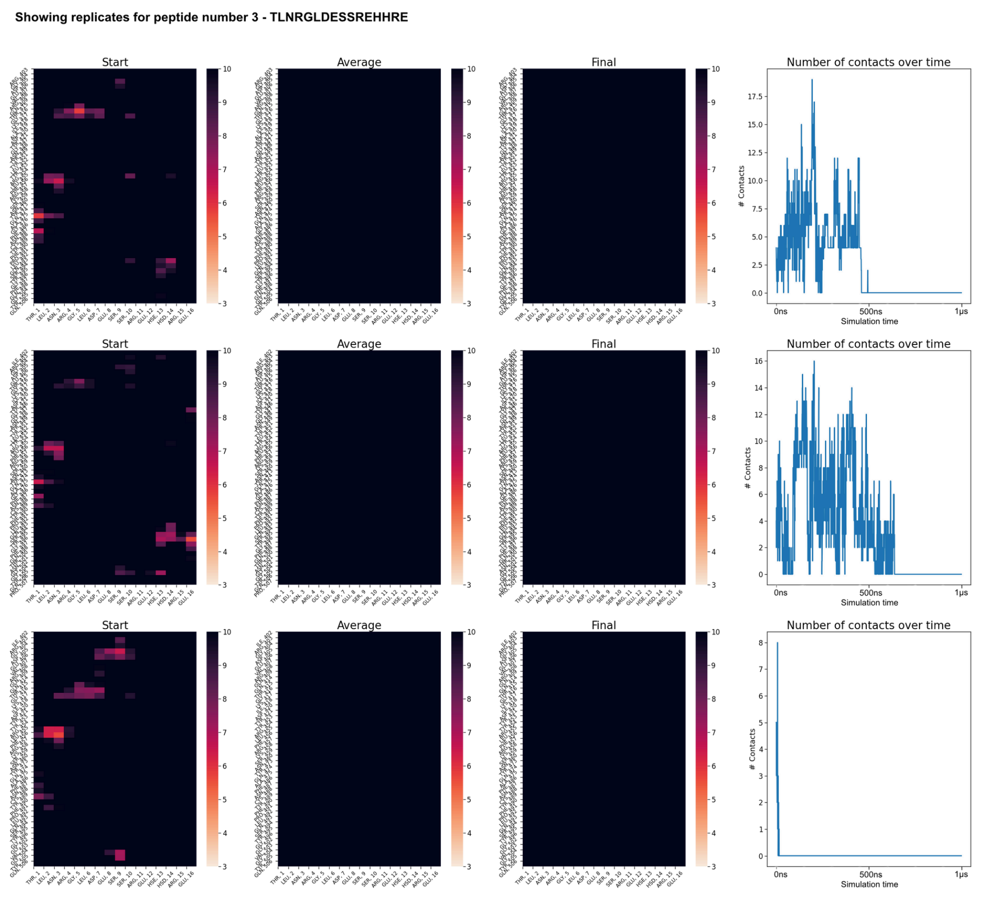
Showing replicates for peptide number 3 - TLNRGLDESSREHHRE
Start
Average
Final
Number of contacts over time
Simulation time

D

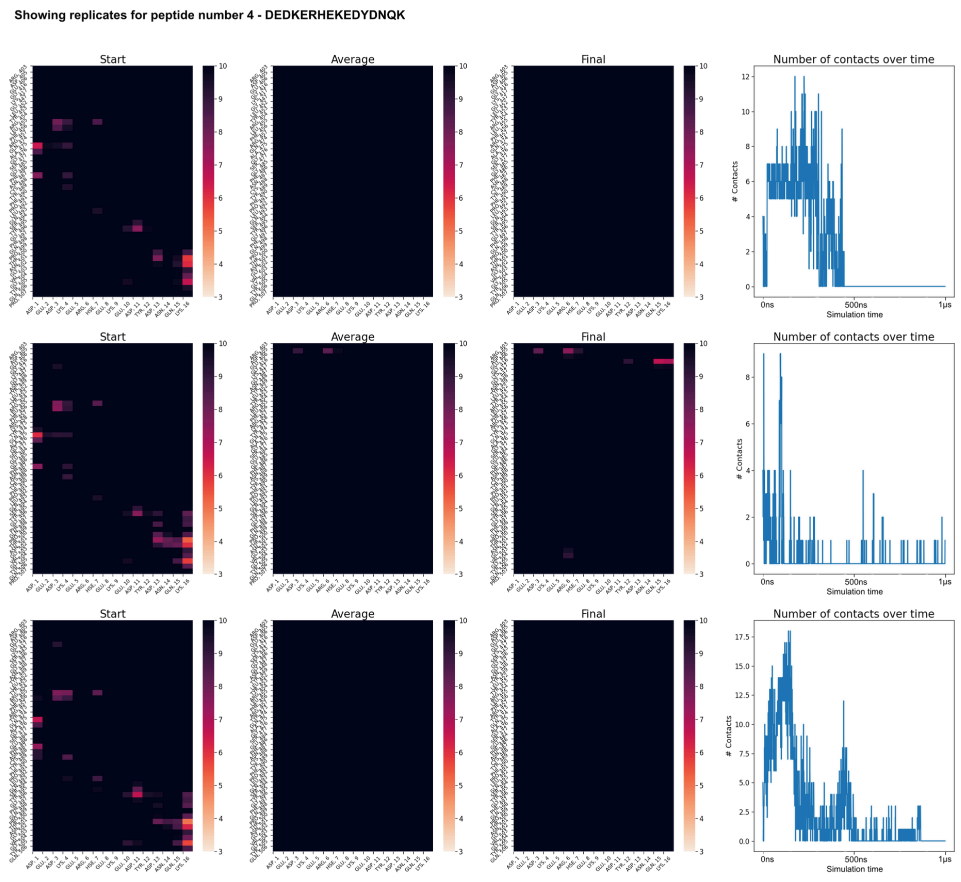
Showing replicates for peptide number 4 - DEDKERHEKEDYDNQK
Start
Average
Final
Number of contacts over time
Simulation time

E

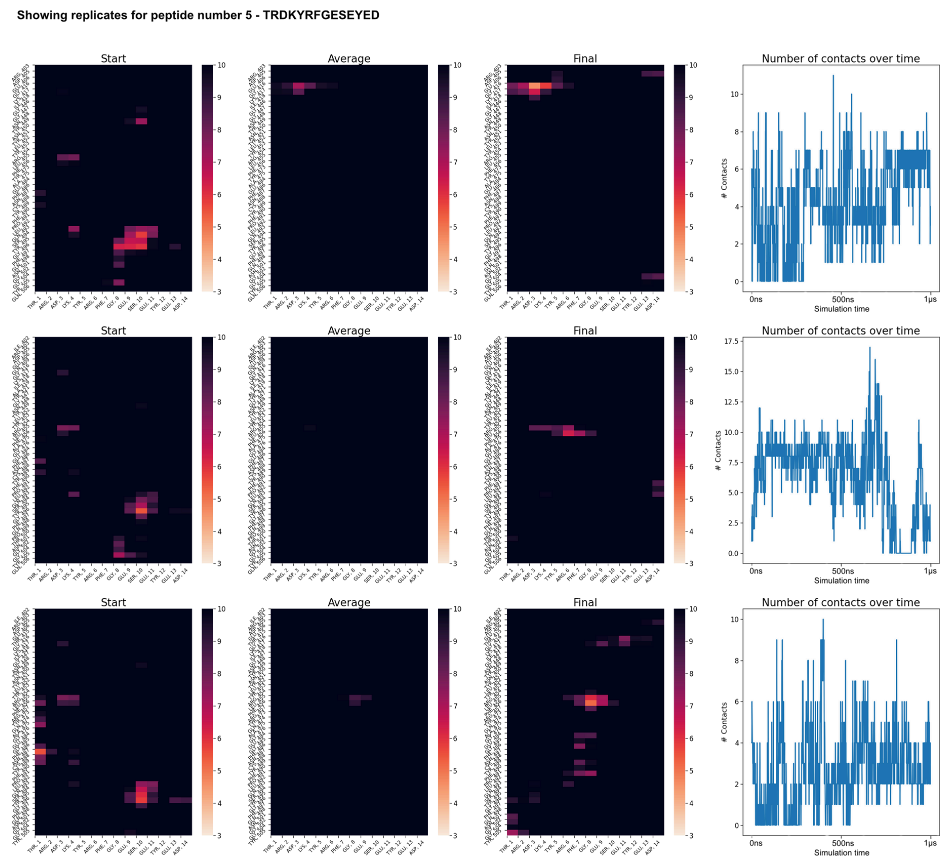
Showing replicates for peptide number 5 - TRDKYRFGESEYED
Start
Average
Final
Number of contacts over time
Simulation time

F

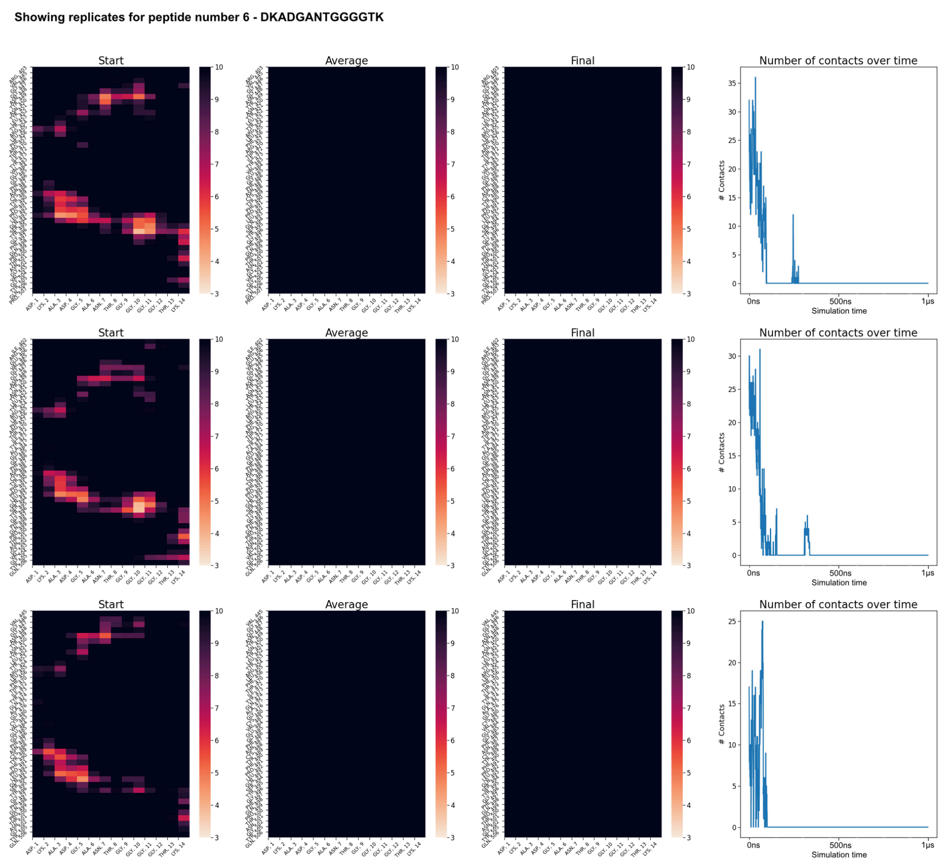
Showing replicates for peptide number 6 - DKADGANTGGGGTK
Start
Average
Final
Number of contacts over time
Simulation time

G

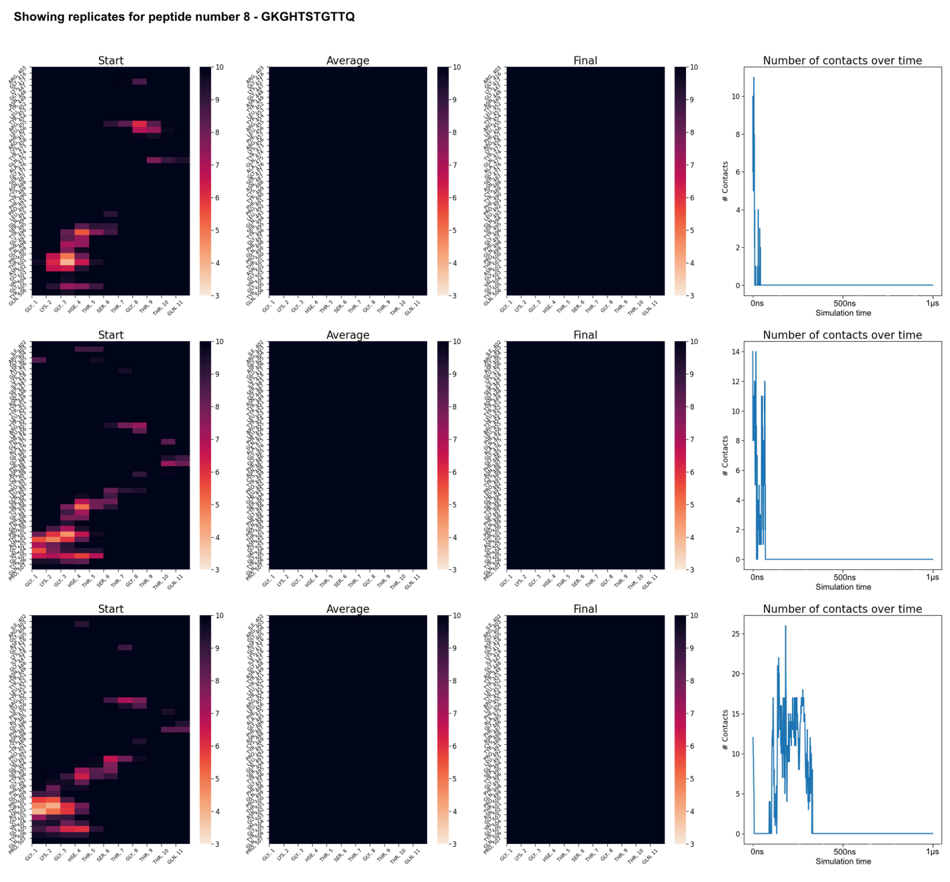
Showing replicates for peptide number 8 - GKGHTSTGTTQ
Start
Average
Final
Number of contacts over time
Simulation time

H

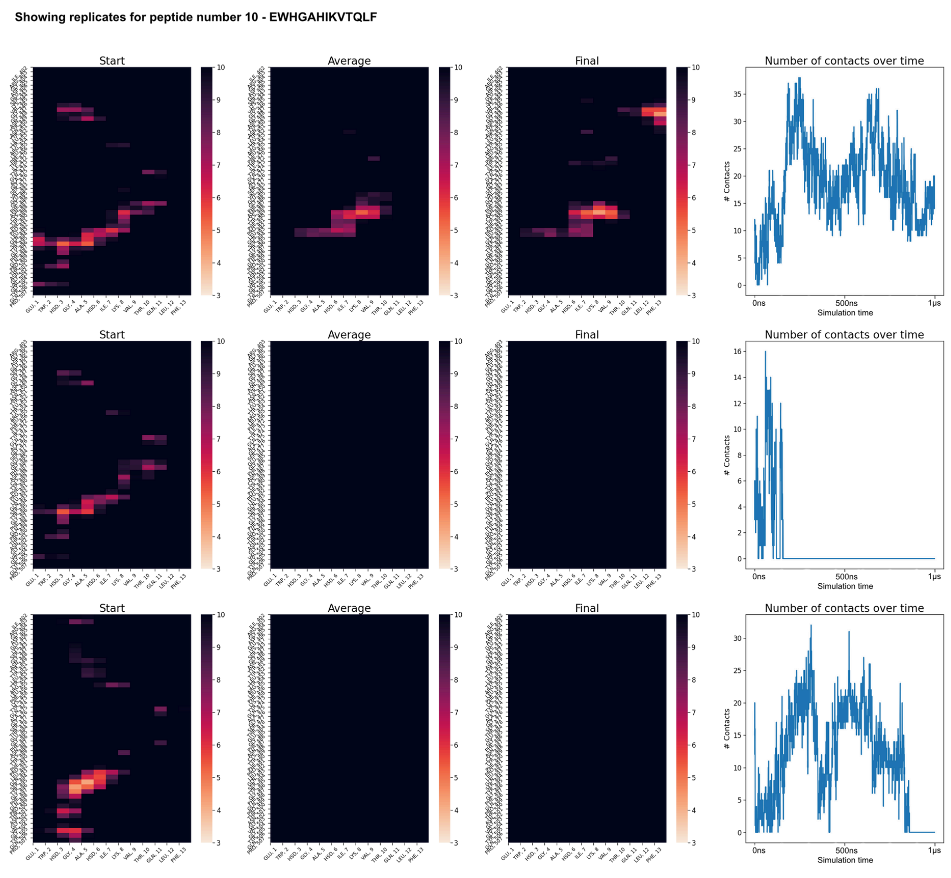
Showing replicates for peptide number 10 - EWHGAHIKVTQLF
Start
Average
Final
Number of contacts over time
Simulation time

I

Showing replicates for peptide number 11 - QDDTQEDKDRHLKDEIYK

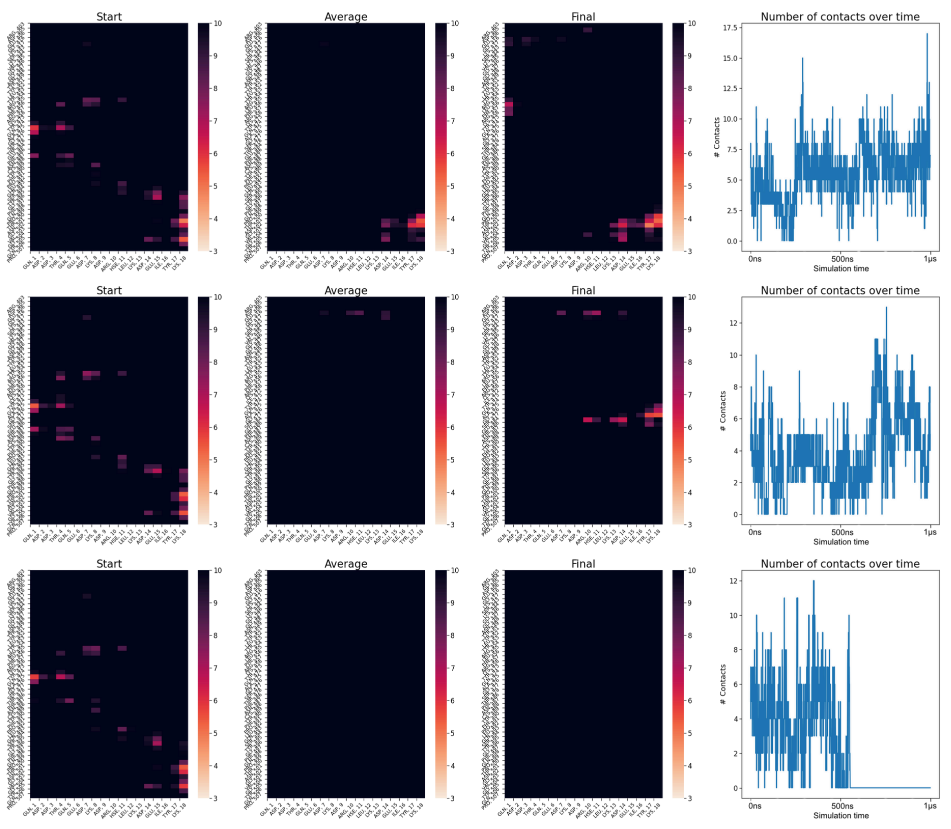

J

Showing replicates for peptide number 12 - QRFSEERYRAWVSHEND

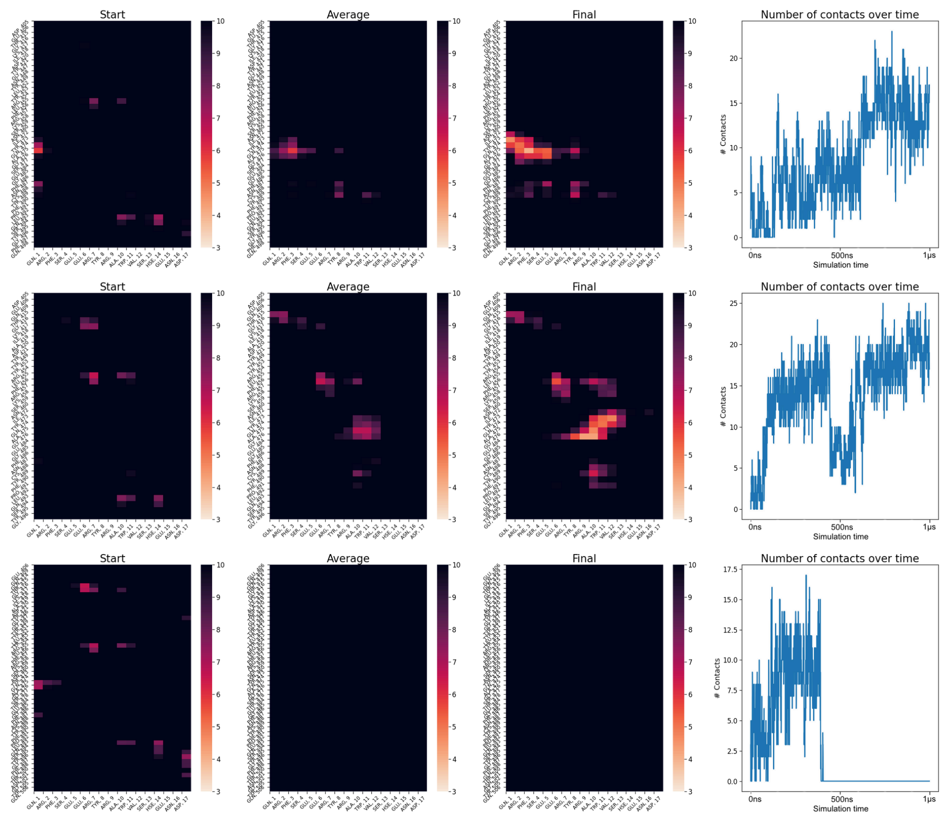

K

Showing replicates for peptide number 13 - QLGDLHRDRLGEENNRQ

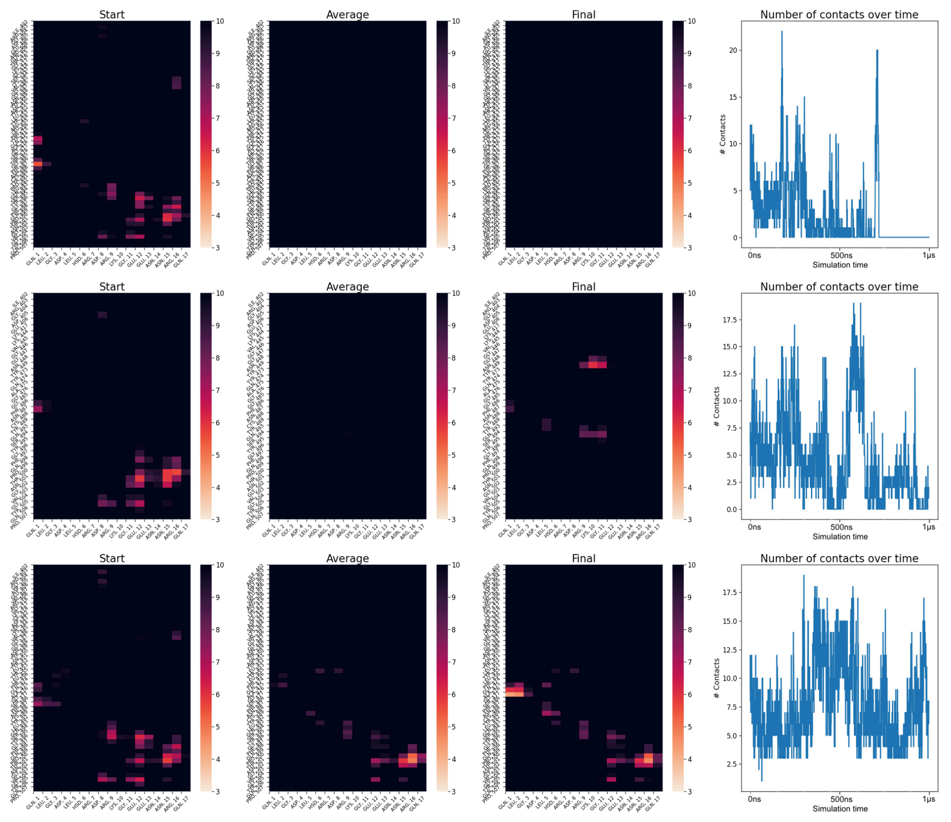

L

Showing replicates for peptide number 14 - QEQTERDKRQHEKDSDWYQ

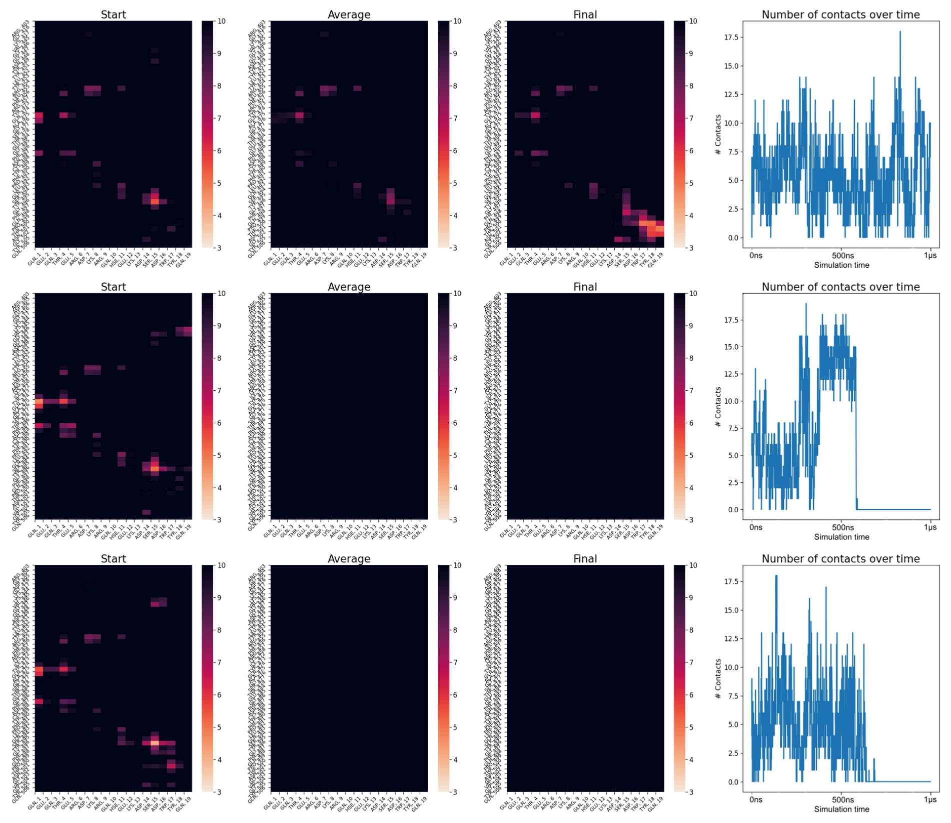

M

Showing replicates for peptide number 15 - TDEDKKYH

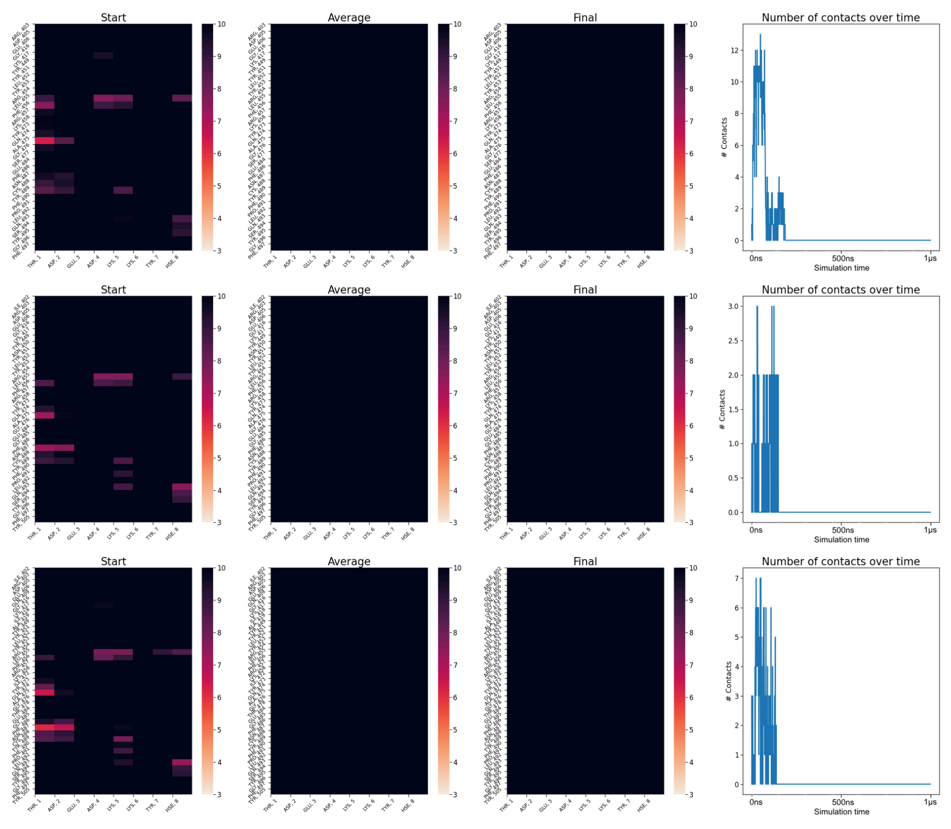

N

Showing replicates for peptide number 16 - TDAGKDGWADHIYHRQY

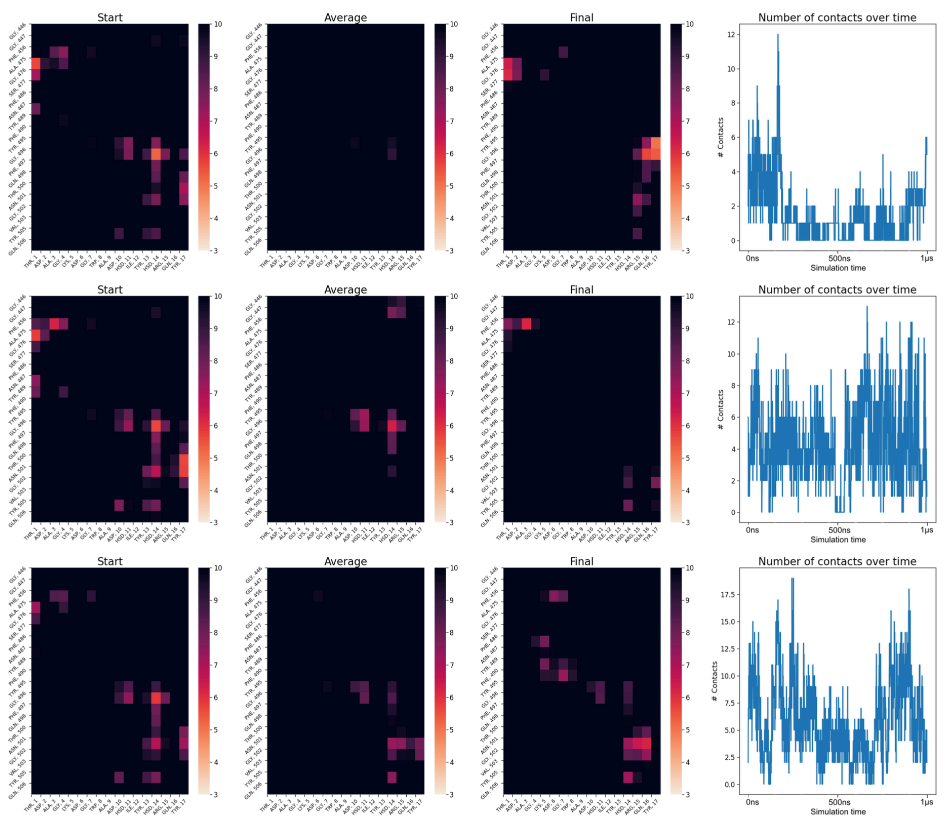

O

Showing replicates for peptide number 17 - TSDDFAEEHWKAHAGAYKL

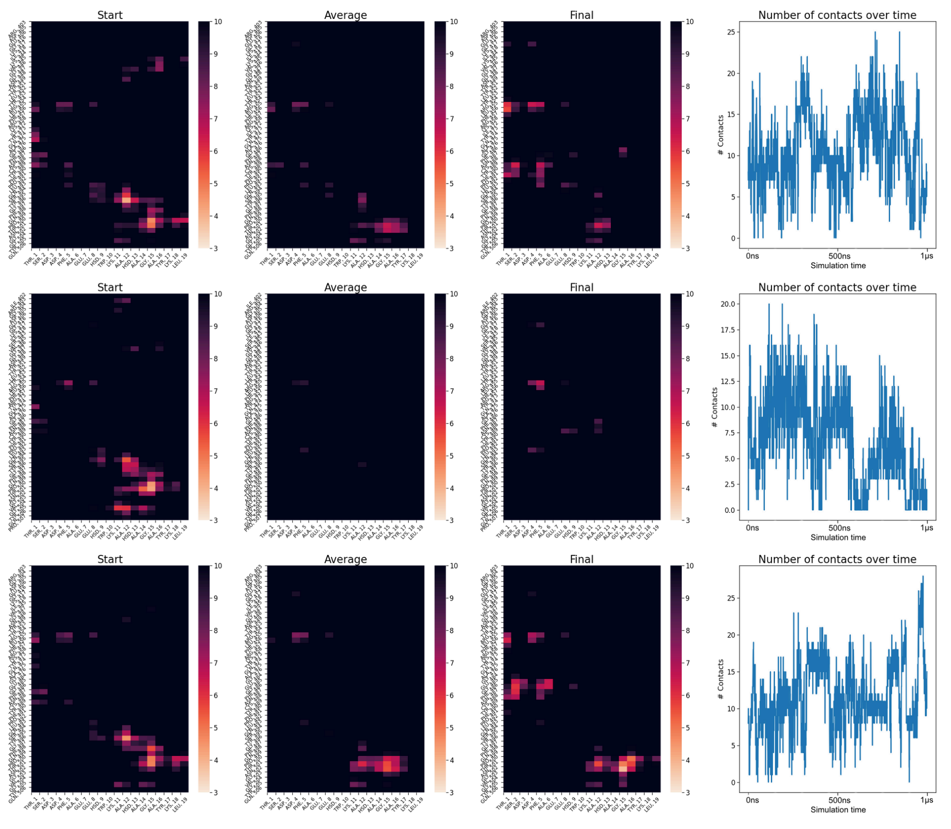

P

Showing replicates for peptide number 18 - NEDKNRHGEASYGNQYG

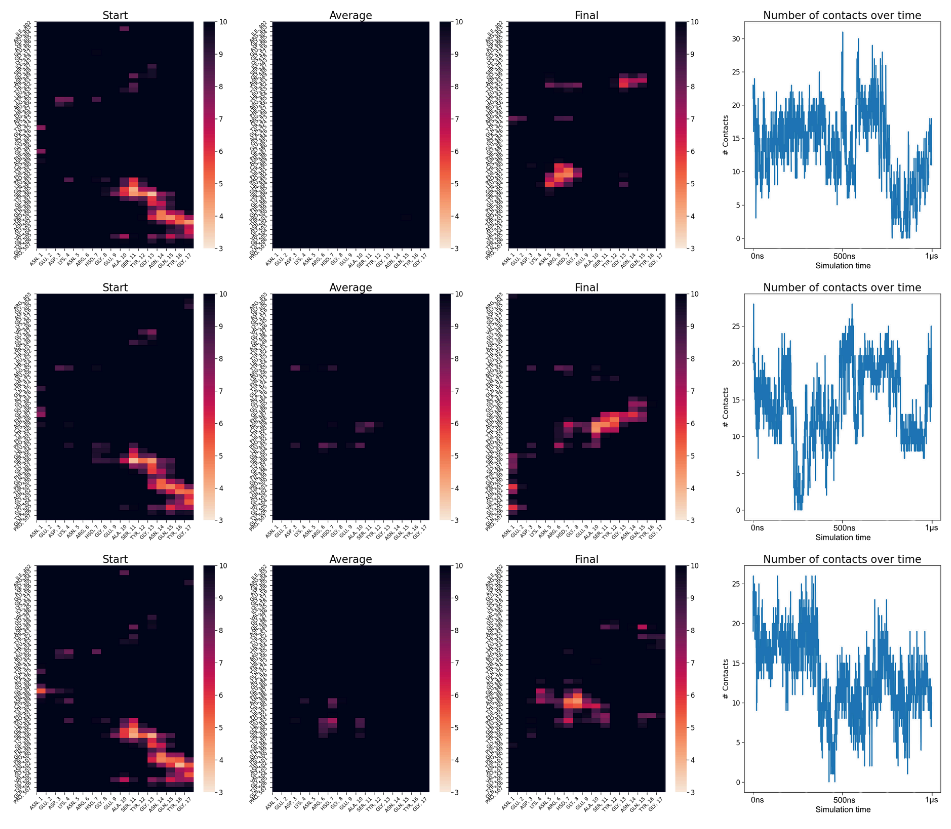

Q

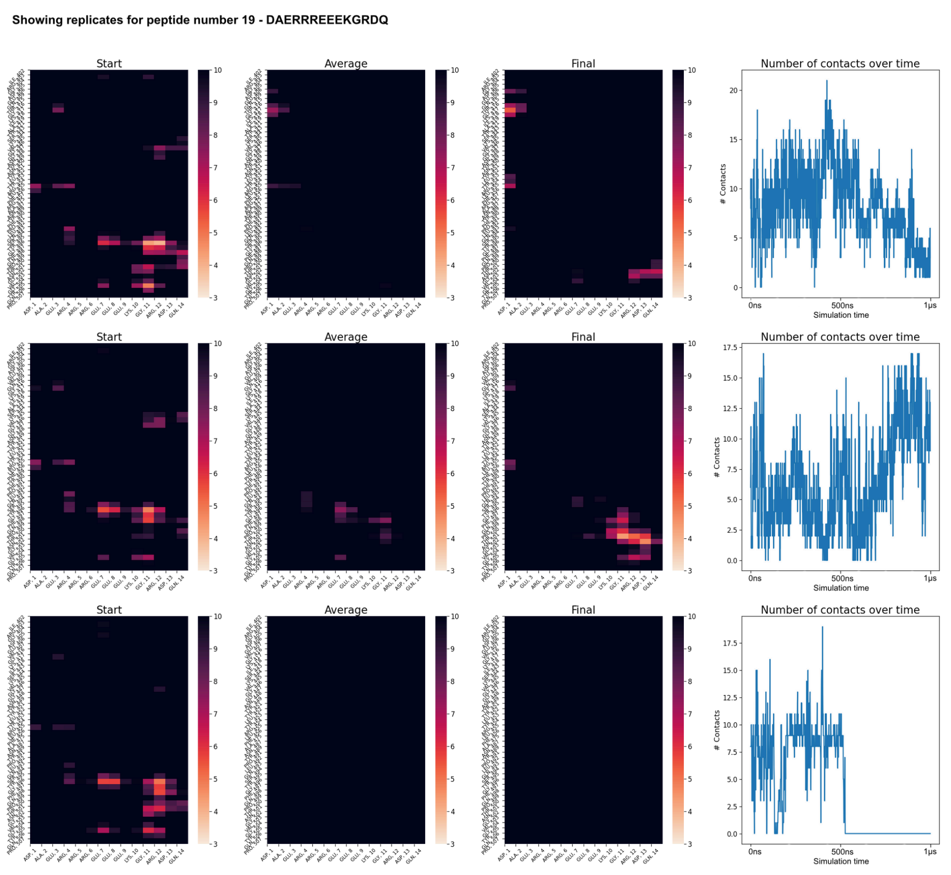

R

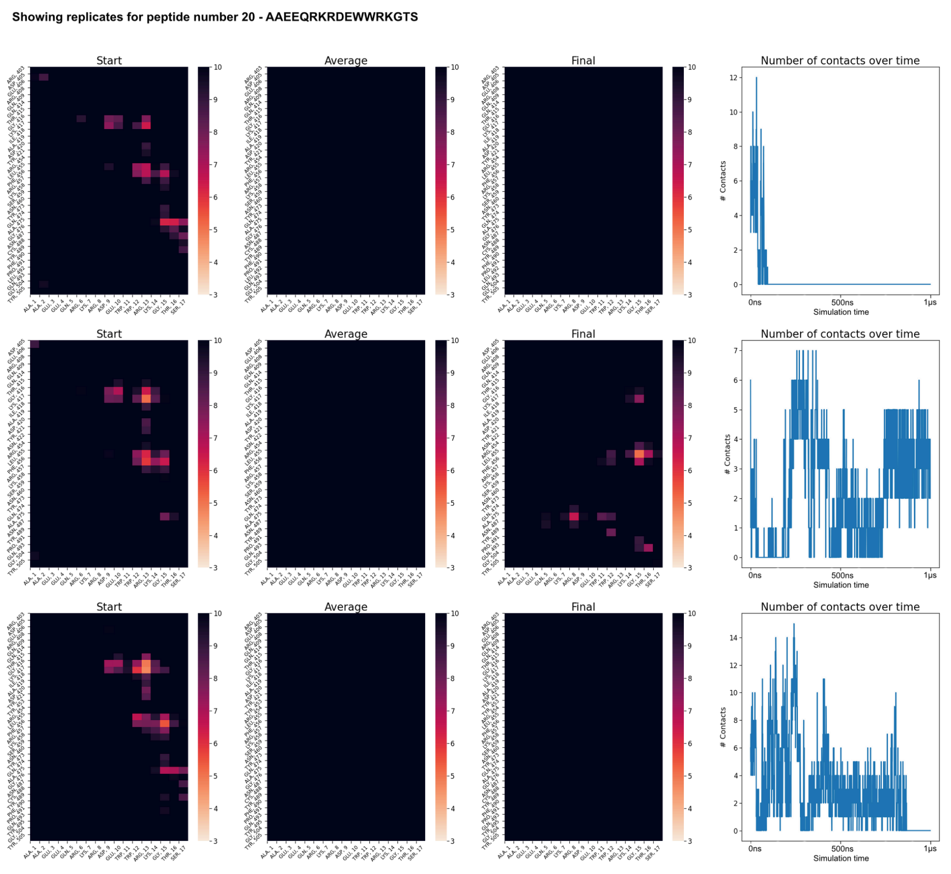

Supplementary Figure S1: Binding Interaction Summary for PEP1–PEP20 from Molecular Dynamics (MD) Simulations.

Each row represents data for one peptide (PEP1–PEP20), except for PEP7 and PEP9, for which the simulation crashed. The simulations were performed in triplicate. For each replicate, the upper panels illustrate binding interactions between the peptide and the SARS-CoV-2 RBD during the first, average, and final timesteps. The lower panel in each replicate shows the time evolution of the number of contacts (distance <7 Å) throughout the simulation. The interpretation of the color gradient is the same as described in Figure 1 of the article. This layout provides a comprehensive overview of interaction dynamics across all replicates for each peptide.
